# Computational Synthetic Biology Enabled through JAX: A Showcase

**DOI:** 10.1021/acssynbio.4c00307

**Published:** 2024-09-04

**Authors:** Olivia Gallup, Kirill Sechkar, Sebastian Towers, Harrison Steel

**Affiliations:** University of Oxford, Department of Engineering Science, OX1 3PJ Oxford, U.K.

**Keywords:** synthetic biology, JAX, computational, modeling, simulation, machine learning

## Abstract

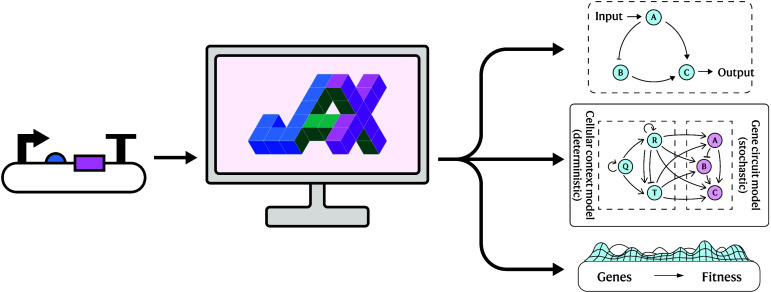

Mathematical modeling is indispensable in synthetic biology but remains underutilized. Tackling problems, from optimizing gene networks to simulating intracellular dynamics, can be facilitated by the ever-growing body of modeling approaches, be they mechanistic, stochastic, data-driven, or AI-enabled. Thanks to progress in the AI community, robust frameworks have emerged to enable researchers to access complex computational hardware and compilation. Previously, these frameworks focused solely on deep learning, but they have been developed to the point where running different forms of computation is relatively simple, as made possible, notably, by the JAX library. Running simulations at scale on GPUs speeds up research, which compounds enable larger-scale experiments and greater usability of code. As JAX remains underexplored in computational biology, we demonstrate its utility in three example projects ranging from synthetic biology to directed evolution, each with an accompanying demonstrative Jupyter notebook. We hope that these tutorials serve to democratize the flexible scaling, faster run-times, easy GPU portability, and mathematical enhancements (such as automatic differentiation) that JAX brings, all with only minor restructuring of code.

## Introduction

Mathematical modeling is an essential stepping stone on the road to rendering biology a true engineering discipline.^1^ Since the seminal works on the repressilator^2^ and the genetic toggle switch,^3^ a plethora of software tools have emerged to support such model-driven design.^4−9^ As an example, software tools, including PyTorch, Tensorflow, and JAX developed largely for AI research have been leveraged to tackle challenges in computational biology. Their advanced handling of matrix computation, just-in-time (JIT) compilation, and integration with graphical processing units (GPUs) greatly enable and speed up complex modeling tasks.^10−13^ JAX in particular shows promise for broad mathematical applications, as it requires the least setup for computations on GPUs compared to PyTorch, Tensorflow, MATLAB (and MLX), or Julia, reads and behaves just like the popular Python mathematical package NumPy, and within synthetic biology has recently been used for simulating SBML models.^7,14^ Its three flagship functions for parallelization (jax.vmap), automatic differentiation (jax.grad), and JIT compilation (jax.jit) can easily wrap existing NumPy-based functions (jax.numpy), making JAX especially amenable to speeding up and scaling up data science workflows alongside a broad array of mathematical modeling.

**Figure 1:**
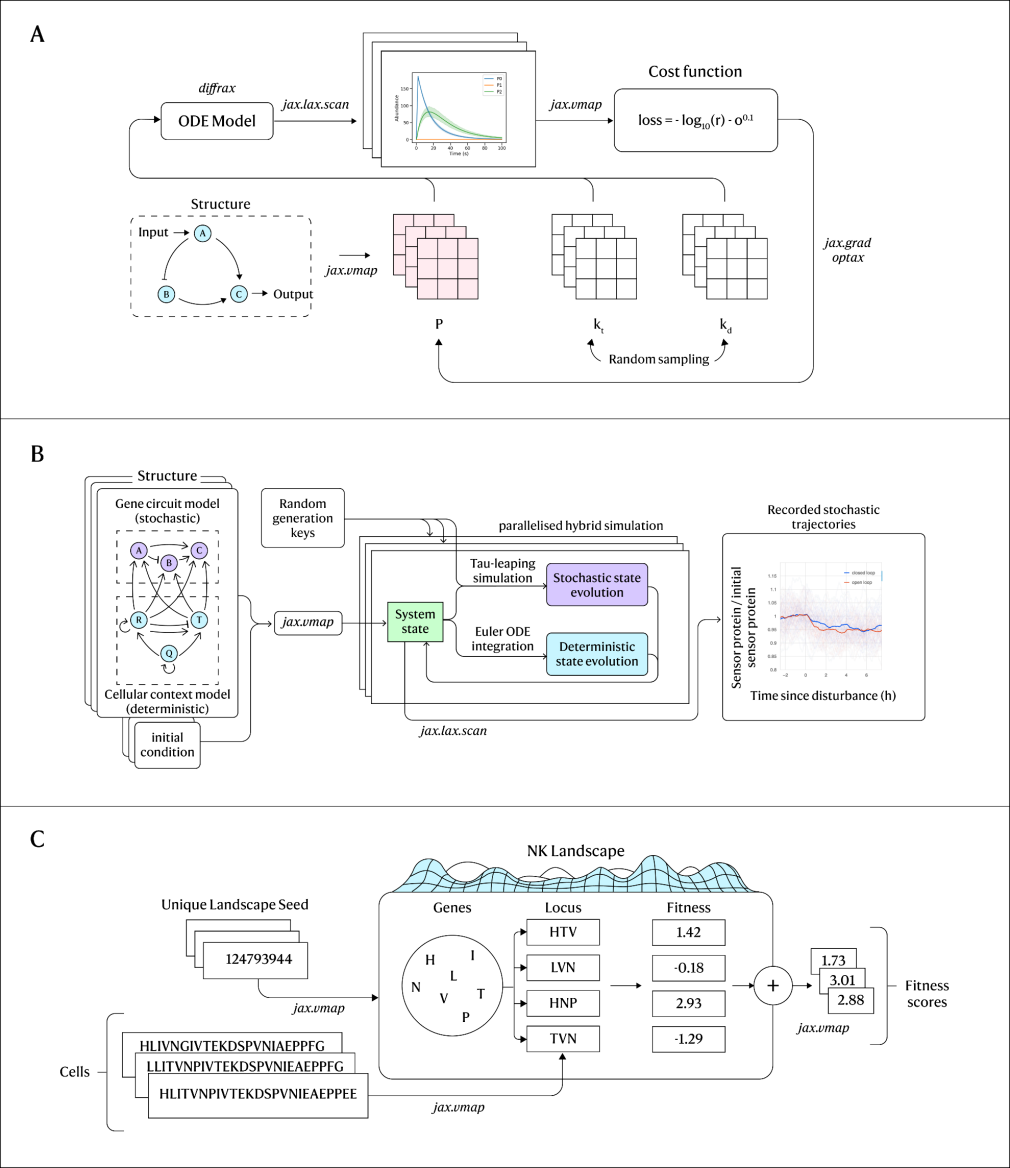
Overview of model structure for showcases. (A) Simulating a differential equation model to search a large parameter space for effective circuit parameters. (B) Simulating cell models using a combination of deterministic and stochastic state evolution allows large models with fast reaction rates to be simulated over long time-periods. (C) Statistical models of evolution such as the simulated NK landscape significantly benefit from parallelization in JAX.

In this work, we present three showcases that use common computational biology modeling approaches and leverage the JAX suite of tools and supporting packages. In each showcase, we describe the problem under investigation, the construction of models, and the structure of the implementation, summarized in Figure 1. All showcases are tied together through the goal of designing a biological system that can fulfill a desired function.

## Results and Discussion

### Showcase 1: Automatic Differentiation and Optimization of Gene Circuits

As an introduction, we will first showcase how to simulate a dynamic differential-equation system and optimize its parameters (Figure 1a). Let us consider the case of a system of interacting genes, for example a genetic circuit where transcription factors control the expression of a fluorescent reporter protein by modulating the rate of transcription. Depending on the network topology and interaction rates between genes and proteins, biological motifs emerge that can exhibit a range of possible stable states, oscillations, or adaptation to perturbations.^2^ The latter is an important engineering goal as biological systems must mediate a variety of perturbations that can potentially derail the intended functionality of a circuit. Our objective is to find a set of parameters for the genetic circuit that will enable the system to adapt to perturbations.

Optimizing such a system using parallelization and automatic differentiation can offer significant advantages when compared to previous approaches. In Ma and Tang et al. 2009,^15^ a large survey of circuit topologies and parameter strengths was carried out to identify motifs that were a prerequisite for adaptation by running individual simulations in a brute force parameter sweep. Later works^16^ have since confirmed and expanded upon the proposed adaptation prerequisites through proofs to guarantee viable topologies. Nevertheless, practically implementing a parameter search on a candidate biological design may require the evaluation of multiple topologies to choose between preferable local maxima or the constraint of some parameters that have already been predetermined. GeneNet^17^ employs automatic differentiation for optimizing a circuit to a given target functionality in PyTorch. We will purposely use a very similar approach here to retain comparability but employ JAX and several of its supporting libraries for numerical approximation and optimization, including Diffrax and Optax, to retain the benefits of flexible parallelization, automatic differentiation, and JIT compilation. For a more detailed description of the problem setting, see 1 Supplementary Section 1. The model structure, loss functions, and relevant variables are described in the full walk-through included in the notebook in this repository: https://github.com/olive004/optimising_gene_circuits.

In this relatively simple example, we initialize a set of circuits randomly and optimize each one individually by leveraging the jax.vmap function without rewriting the single-circuit simulation functions. After simulating the dynamics of each circuit, each optimization loop is run by the jax.lax.scan function which is further sped up by JIT compilation with a jax.jit wrapper, again without rewriting any core code. The dynamics are scored through the objective function, which is wrapped with the jax.grad in order to automatically differentiate with respect to the circuit parameters, which are then updated using the Adam optimizer available through Optax (optax.adam). Because a function only has to contain JAX primitives in order for it to be wrapped in the described manner, and because the vast majority of NumPy functions are available in jax.numpy, the outsized benefits gained from GPU-based parallelization, JIT compilation, and automatic differentiation can be integrated easily into a typical data science workflow for systems and synthetic biology.

### Showcase 2: Stochastic Cell Model Simulations

Dynamic simulation of genetic circuits is an integral part of design and prototyping in synthetic biology. However, biology is not deterministic. Extending from Showcase 1, taking stochasticity into consideration is crucial, as randomness is a central feature of living systems and may give rise to behaviors that cannot be explained through deterministic simulations.^18^ For example, deterministic simulations are not suited to identifying bistability, noise attenuation, and genetic glitches.^19^ We explore the benefits of a JAX implementation of a Tau-leaping algorithm,^20^ which is based on the Gillespie algorithm for approximating stochastic systems and is amenable to large models thanks to its increased simulation efficiency.

To investigate the potential of JAX-accelerated simulation of stochastic processes, we apply it to resource-aware cell models that consider synthetic circuit genes alongside the genes governing the metabolism of the cell that hosts them, as well as external factors such as the density of energy sources in the growth medium and the presence of antibiotic compounds (see Figure 1b). This context-aware modeling is motivated by observations that synthetic genes are affected (e.g., in terms of expression level) both by one another *and* by the host cell’s state^10,21^ (for more problem setting details, see Supplementary Section 2). However, this has typically been difficult to computationally investigate, as models that incorporate large numbers of species/interactions and/or reactions with very fast rates quickly become computationally unwieldy. In this case, efficient means of simulation drastically alter the usability of a model, especially because these complex memory-intensive models must be run repeatedly (i.e., as Monte Carlo simulations) to generate accurate distributions of possible outcomes and behaviors.

**Figure 2:**
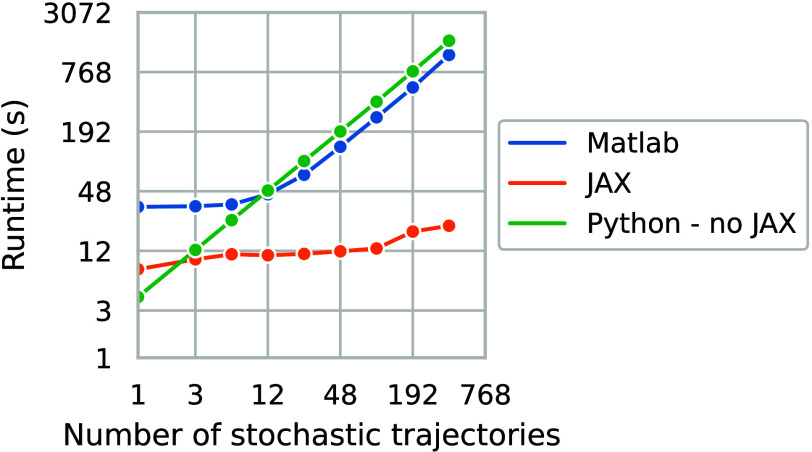
Log–log plot of the time taken to simulate 1 min of the cell model’s stochastic dynamics using Matlab and JAX implementations.

Fortunately, the vectorization and GPU parallelization described in Showcase 1 allow for a dramatic speed up in stochastic simulations of these larger-scale models. This can be seen upon running this guiding notebook (https://github.com/KSechkar/rc_ecoli_jax/blob/main/jax_implementation/sim_script.ipynb) to obtain 48 sample trajectories of a hybrid resource-aware cell model,^10^ which comprises 11 stochastic variables simulated with a tau-leaping algorithm and 6 variables simulated deterministically using the Euler method (as their stochastic fluctuations are averaged out in the cell^22^). Running the model’s JAX-based implementation on a GPU (NVIDIA GeForce RTX 4090) is 15 times faster than running the analogous MATLAB script on the same PC, even when the latter leverages parallelization to employ all 12 of the two-threaded cores of an Intel(R) Xeon(R) w5-2455X CPU. As the number of simulated trajectories increases, the JAX implementation’s runtime scales sublinearly (suggesting we are still not fully utilizing the GPU’s capacity), unlike MATLAB simulations that can only run in sequence without a significant rewrite after the CPU’s parallelization capacity is reached at 12 trajectories. Meanwhile, although the model’s implementation in Python without JAX is faster to simulate a single trajectory, its inability to leverage parallelization on the GPU means that its linearly scaling runtime exceeds that of JAX code for as few as three trajectories being simulated (Figure 2).

Compute-heavy cases like these tend to be skipped in the biodesign workflow by synthetic biologists as the time and effort investments required to set up such simulations are typically too high to consider on a routine basis. However, JAX allows for code that is both efficient and easy to use and reuse—for instance, this walkthrough notebook (https://github.com/KSechkar/rc_ecoli_jax/blob/main/jax_implementation/howto_example.ipynb) outlines the steps for repurposing the resource-aware cell modeling framework discussed above for the simulation of another gene circuit hosted by the cell. JAX’s flexibility and user-friendliness therefore not only integrate well into existing code but also offer insights that are otherwise only available to those with sufficient coding skills and narrow the gap between dry and wet lab scientists.

### Showcase 3: Directed Evolution Strategies

In our final showcase (Figure 1c), we consider population-level simulations, specifically for predicting the effects of directed evolution policies.^23^ For simulating a population of cells undergoing evolution, we can use the NK model^24^ as an example. The titular parameter *N* represents the size of the dictionary defining each evolutionary unit, in this case the vector length of each gene, and *K* defines the ruggedness of the fitness landscape. Other parameters include the population size, choice of selection strategy, chance of a mutation occurring within a gene, how many genes there are in each cell, and how many steps are run in each iteration (see Figure 1c). This framework allows the exploration of the NK model parameters and comparison to real evolutionary landscapes, such as the previously mapped GB1 protein landscape.^25^ For more detail on the model, readers are referred to the methods section of Towers and James et al. 2024.^23^

The NK landscape naturally lends itself to parallelization. Each of the cells in a population may be simulated at the same time, and each “fitness component” of a cell may also be calculated simultaneously.

The automatic differentiation capabilities of JAX are in this case secondary to the ease of parallelization of the entire workflow. A population of cells subjected to directed evolution may be efficiently simulated in parallel. By distribution of the computation of fitness scores for each cell, this efficiency can be retained even for large populations. By making each function JAX-compatible, simulation runs can be layered simply by wrapping functions with the vectorization function *jax.vmap*. Functions that previously did calculations only for single numbers can now work across matrices. This makes it easier to control and vary many variables to identify overall trends, such as changing the selection criteria in each generation or using different distributions of genes. The key JAX benefits highlighted by this example are flexibility and scalability. While PyTorch also has a JAX-inspired vmap function, its use supported cases are much more limited, even more so for NumPy’s vectorization function.

Assessing the ability of evolution policies to elicit better performers in a population requires many repeat simulations with random initializations. In this case, not only is there randomness in the trajectory each cell or gene takes as the population evolves (for example due to random mutations applied in each generation) but also the underlying equations, such as the selection method or fitness landscape properties themselves, *also* contain randomness, changing with every new simulation. Unlike PyTorch or MATLAB, JAX treats random keys as central to most functions and ensures independent seeding by allowing keys to be split based on combinations of keys and function outputs. Biases that might be introduced accidentally when sampling distributions repeatedly or in parallel can thereby be mitigated. The code guiding this example can be found in this notebook (https://github.com/nesou2/direvo_sim).

## Conclusion

We have presented the benefits of integrating JAX into new and existing systems and synthetic biology workflows. We compare the ease of use and implementation compared to other mathematical coding frameworks like PyTorch and MATLAB. We show that JAX democratizes running large-scale simulations with GPUs through parallelization, JIT compilation, and close compatibility with existing packages, reducing the need for a cumbersome rewrite of Python modeling scripts. Readers are encouraged to explore the GitHub repositories linked in the article. We hope that these examples and guiding code will facilitate the adoption of JAX vectorization and automatic differentiation into synthetic biology modeling efforts both within and beyond the scope of applications discussed in the present article.

## Supporting Information Available

Supplementary notes for showcases 1 and 2 (PDF)

## Abbreviations

CPU: Central processing unit
GPU: Graphical processing unit
JIT: Just-in-time
