# Supplementary material for: Computational Synthetic Biology Enabled through JAX: A Showcase
Source: ACS Synth Biol. 2024 Sep 4;13(9):3046–50. doi: 10.1021/acssynbio.4c00307 (PMC11421211; doi:10.1021/acssynbio.4c00307)
Supplement: Supplementary file 1 — sb4c00307_si_001.pdf [file sb4c00307_si_001.pdf]

Supporting Information for 'Computational synthetic biology enabled through JAX: a showcase'

Olivia Gallup*,* Kirill Sechkar,* Sebastian Towers,* and Harrison Steel*

University of Oxford, Department of Engineering Science, OX1 3PJ, UK

E-mail: olivia.gallupova@eng.ox.ac.uk; kirill.sechkar@queens.ox.ac.uk; sebastian.towers@eng.ox.ac.uk; harrison.steel@eng.ox.ac.uk

Supplementary notes

Supplementary note for showcase 1: Automatic differentiation and optimisation of gene circuits

In order to make the first example JAX showcase as straightforward to understand as possible, we focus on a system similar to Hiscock et. al. 2019.[1] As in the cited work, we set up a circuit with n nodes (proteins) with randomly initialised transcription k_t and degradation rates k_d. Here, we sample from a normal distribution with k_t mean = 0.01, k_d mean = 0.07, and standard deviation of 0.01. The interactions between proteins are defined as an n-by-n matrix P and the amounts of each circuit species is represented in a n-by-1 vector x, giving the following ODE equation:

$$dx = k_t + params * x - k_d * x \tag{S1}$$

In order to optimise for adaptation to a perturbation, a step response is added as a signal input and the system's response can be assessed in the objective function. Using the above equation, each optimisation loop is initialised with x = 0. The step input is then introduced by adding a fixed amount 100 at $t = 0$ to a species x_{sig} that we define as the signal species, and the system is simulated for time interval t_1. The robustness of the circuit can be evaluated using the metrics of sensitivity and precision, which have been defined in Ma and Tang et al. 2009[2] as a measure of how responsive a system is to a perturbation (sensitivity) and how closely it can return to its pre-perturbed state (precision), defined as following:

$$S = \left| \frac{O_{peak} - O_i}{I_f - I_i} \right| \tag{S2}$$

$$P = \left| \frac{O_f - O_i}{I_f - I_i} \right|^{-1} \tag{S3}$$

where S is sensitivity, P is precision, I is the input, and O is the output. I_i and I_f represent the initial and final inputs, while O_i and O_f represent the initial and final outputs.r The cost function for the optimisation can thus be defined as a weighted sum of sensitivity and precision. To prevent the trivial case arising where the circuit parameters tend towards zero, the overshoot is also added to the loss (scaled and negative). The cost is thus defined through the following functions:

$$r = frac(w_s * S)(w_p * P) \tag{S4}$$

$$o = |y_{peak} - y_{final}| \tag{S5}$$

$$l = -(log(r) + o^{0.1}) \tag{S6}$$

where r is robustness, o is the overshoot, w_s and w_p are the weights for sensitivity and robustness respectively, and l is the cost. For optimising the system, we backpropagate from the cost function to find optimal parameters.

Supplementary note for showcase 2: Stochastic cell model simulations

Cell model definition and simulation algorithm

To demonstrate the use of JAX for stochastic model simulations, we considered the example of a coarse-grained resource-aware cell model proposed in Sechkar and Steel et al. 2024.[3] While an in-depth explanation of this model's assumptions, equations and properties can be found in the cited work, a basic discussion of its definition is reproduced here.

A hybrid approach is used to simulate the behaviour of an *E. coli* cell hosting a synthetic gene circuit. The expression of the cell's native genes is captured by just a handful of lumped variables, each representing the average dynamics of a large set of abundances of species with similar behaviour and functions. Due to this coarse-graining, stochastic fluctuations of individual variables are averaged out, thereby simulating the host cell's state deterministically. Conversely, each gene in the synthetic gene circuit is treated individually, so the stochasticity of gene expression is expected to significantly affect the dynamics of the circuitry-associated variables.[4]

The dynamics of the host cell X are thus described by the following system of ordinary differential equations (ODEs):

$$\dot{m_a} = c_a\alpha_a\lambda(\epsilon, B) - (\beta_a + \lambda(\epsilon, B))m_a \tag{S7}$$

$$\dot{m_r} = F_r(T)\cdot c_r\alpha_r\lambda(\epsilon, B) - (\beta_r + \lambda(\epsilon, B))m_r \tag{S8}$$

$$\dot{p_a} = \frac{\epsilon(t^c)}{n_a}\cdot\frac{m_a/k_a}{D}R - \lambda(\epsilon, B)\cdot p_a \tag{S9}$$

$$\dot{R} = \frac{\epsilon(t^c)}{n_r}\cdot\frac{m_r/k_r}{D}R - \lambda(\epsilon, B)\cdot R \tag{S10}$$

$$\dot{t^c} = \nu(t^u, \sigma)\cdot p_a - \epsilon(t^c)\cdot\left(B - R\frac{\sum_{x_l\in X} m_{x_l}/k_{x_l}}{D}\right) - \lambda(\epsilon, B)\cdot t^c \tag{S11}$$

$$\dot{t^u} = \psi(T)\cdot\lambda(\epsilon, B) - \nu(t^u, \sigma)\cdot p_a + \epsilon(t^c)\cdot\left(B - R\frac{\sum_{x_l\in X} m_{x_l}/k_{x_l}}{D}\right) - \lambda(\epsilon, B)\cdot t^u \tag{S12}$$

Here, the cell's genome is partitioned into three classes: ribosomal (r), metabolic (a) and housekeeping (q). Since the mass fraction of housekeeping genes is always $\overline{\phi}_q = 0.59$, we can avoid modelling it explicitly. For the remaining two classes, we consider their mRNA concentrations m_r and m_a and protein concentrations p_r and p_a. By analogy, the mRNA and protein abundances for any synthetic gene x_l in the set $X = \{x_1,\ x_2,\ \dots x_l\}$ expressed by the cell are denoted as m_{x_l} and p_{x_l}. Meanwhile, t^c and t^u represent the concentrations of aminoacylated and free tRNA concentrations in the cell, respectively. The extent of competition for ribosomes in the cell is captured by the "resource competition denominator" D (Equation (S13)). The meaning, definitions and values of all other parameters and functions appearing in the ODEs are displayed in Tables S1 and Tables S2 copied with minimal changes from Supplementary Tables S1 and S2 in Sechkar and Steel et al. 2024.[3]

$$D = 1 + \frac{1}{1 - \overline{\phi}_q} \sum_{j \in \{a,r\} \cup X} m_j / k_j \tag{S13}$$

For a synthetic circuit gene $x_l \in X$ with mRNA and protein counts m_{x_l} and p_{x_l}, we list the considered stochastic reactions below. In these expressions, F_i is the gene-specific transcription regulation function and all other parameters and functions have the same meaning and form as for the host cell model ODEs.

- **Transcription** at rate $F_{x_l} \alpha_{x_l} c_{x_l} \lambda \cdot \left(\frac{n_{x_l}}{25}\right)^{-1}$. Increases m_{x_l} by $\frac{n_{x_l}}{25}$
- **mRNA degradation** at rate $\beta_{x_l} m_{x_l} \cdot \left(\frac{n_{x_l}}{25}\right)^{-1}$. Decreases m_{x_l} by $\frac{n_{x_l}}{25}$
- **mRNA dilution** at rate $\lambda m_{x_l} \cdot \left(\frac{n_{x_l}}{25}\right)^{-1}$. Decreases m_{x_l} by $\frac{n_{x_l}}{25}$
- **Translation** at rate $\frac{\epsilon}{n_{x_l}} \cdot \frac{m_{x_l}/k_{x_l}}{D} \cdot R$. Increases p_{x_l} by 1, decreases t^c by n_{x_l} (charged tRNAs being used up), increases t^u by n_{x_l} (uncharged tRNAs produced)
- **Protein dilution** at rate λp_{x_l}. Decreases p_{x_l} by 1

Additionally, if RNA transcripts of gene $x_{l,1}$ and gene $x_{l,2}$ can bind to each other and

thereby mutually annihilate, an additional stochastic annihilation reaction is introduced, where θ is the RNAs' mutual annihilation rate:

- **mRNA annihilation** at rate $\theta m_{l,1} m_{l,2}$. Decreases $m_{l,1}$ by $\frac{n_{l,1}}{25}$ and decreases $m_{l,2}$ by $\frac{n_{l,2}}{25}$

This definition describing the continuous and stochastic parts of the hybrid model allows its simulation on the time interval $t \in (t_{start},\ t_{finish})$ using a tau-leaping Algorithm S1, wherein all model variables are gathered into the vector $\mathbf{v} = (m_a\, m_r\, p_a\, R\, t^c\, t^u\, m_{x_1}\ \dots m_{x_L}\, p_{x_1} \dots p_{x_L})^\top$.

Algorithm S1 Hybrid tau-leaping cell model simulation algorithm.

Set $t = t_{start}$, $\mathbf{v} = \mathbf{v}_{start}$
while $t < t_{finish}$ **do**
    Find the deterministic change in variables $\Delta_{det}\mathbf{v}$ by integrating ODEs (S7)-(S12) over the time interval $(t,\ t + \Delta t)$
    Determine the number of times each stochastic reaction occurred over $(t,\ t + \Delta t)$ by sampling a Poisson distribution with mean equal to $\textit{reaction rate} \times \Delta t$
    Sum the total changes in variables caused by stochastic each reaction over $(t,\ t + \Delta t)$ to obtain $\Delta_{stoch}\mathbf{v}$
    Set $\mathbf{v} = \mathbf{v} + \Delta_{det}\mathbf{v} + \Delta_{stoch}\mathbf{v}$
    for all entries v_j in $\mathbf{v}$ **do**
        if $v_j < 0$ **then**
            Set $v_j = 0$ to avoid negative molecule counts
        end if
    end for
    Set $t = t + \Delta t$
end while

Gene circuit description

The model and algorithm described above allow modelling of the behaviour of an arbitrary gene circuit hosted by a bacterial cell. Here we describe the particular circuit simulated in the showcase provided in this paper, which has been proposed and analysed in Sechkar and Steel et al. 2024[3] and also described the Matlab implementation of the simulation script used as the benchmark for our JAX version.

The circuit is an antithetic integral feedback controller aiming to maintain a constant level of competition for ribosomes in the cell, which is comprised of four genes: the sensor (*sens*), the annihilator (*anti*), the actuator (*act*), and the amplifier (*amp*). The concentration of the constitutively expressed sensor gene protein depends on the number of ribosomes available for translation, and regulates the expression of annihilator RNAs. By binding and mutually annihilating with the constitutively expressed mRNAs of the actuator gene, the annihilator thus implements integral feedback with ribosome availability as the controlled variable. The actuator protein in turn regulates the amplifier gene's expression. By adjusting the abundance of amplifier gene transcripts, the controller is able to manipulate the extent of mRNA competition for the cell's ribosomes, keeping it constant despite disturbances. As an example of such disturbance, we consider the synthetic gene *dist* which becomes active at a time t_{dist}.

The stochastic behaviour of this circuit is therefore simulated according to the previous section, with the set of synthetic gene in the cell being $X = \{sens,\ anti,\ act,\ amp,\ dist\}$ and the mutual RNA annihilation reaction possible for $x_{l,1} = act$ and $x_{l,2} = anti$. The transcription regulation functions are thus

$$F_{sens} = F_{act} = 1 \tag{S14}$$

for the constitutive sensor and actuator gene,

$$F_{anti} = \frac{K_{sens}}{K_{sens} + p_{sens}} \quad \text{and} \quad F_{amp} = \frac{p_{act}}{K_{act} + p_{act}} \tag{S15}$$

for the regulated annihilator and actuator genes, and

$$F_{dist} = \begin{cases} 0 & \text{if } t < t_{dist} \\ (t - t_{dist})/0.1 & \text{if } t_{dist} \geq t < t_{dist} + 0.1 \\ 1 & \text{otherwise} \end{cases} \tag{S16}$$

for the time-dependent disturbance gene. All synthetic gene parameters are given in Table S3, copied with minimal changes from the Supplementary Table S9 in Sechkar and Steel et al. 2024.[3]

Simulation details

In all versions of the code, the tau-leaping step used in the simulation was $\Delta t = 10^{-6}\ h$. As for ODE integration, in the JAX-enabled program it was performed using the Euler method with a time step of $10^{-7}\ h$. In the JAX-free Python, we used the `scipy.integrate.solve_ivp` function with the `RK45` method and relative and absolute tolerances of 10^{-6} and 10^{-9}. In the Matlab version, we employed the `ode15s` solver with the same integration tolerances.

Supplementary Table S1: **Parameters appearing in the cell model ODEs and Supplementary Table S2.**

Parameter	Description	Value	Units[*]
M	Total cell mass (in amino acids)	$1.19 \cdot 10^9$	$aa^{\#}$
σ	Extracellular nutrient quality	0.5	None
$\overline{\phi}_q$	Housekeeping prot. mass fraction	0.59	None
	**Reaction rates**		
ϵ_{max}	Max. translation elongation rate	72,000	$aa^{\#}/h$
ν_{max}	Max. tRNA charging rate	$4,046.9$	h^{-1}
ψ_{max}	Max. tRNA synthesis per unit growth rate	$4.32 \cdot 10^5$	nM
K_ϵ	Michaelis constant for translation elongation	$1,239.7$	nM
K_ν	Michaelis constant for tRNA charging	$1,239.7$	nM
τ	Michaelis constant for ppGpp signalling	1	None
	**Gene expression**		
c_i	Concentration of gene i DNA[‡]	1	nM
α_a	Promoter strength for gene a	$3.945 \cdot 10^5$	None
α_r	Promoter strength for gene r	$4.070 \cdot 10^5$	None
β_i	mRNA degradation rate[‡]	6	h^{-1}
k_i^+	mRNA-ribosome binding rate[‡]	60	$\frac{1}{nM \cdot h}$
k_i^-	mRNA-ribosome dissociation rate[‡]	60	h^{-1}
n_a	Number of amino acids in protein p_a	300	$aa^{\#}/nM$
n_r	Number of amino acids in rib. protein	7,459	$aa^{\#}/nM$

[*] *E. coli* volume is $\approx 10^{-18}$ m^3, so 1 nM is roughly equivalent to 1 molecule/cell.
[#] Amino acid residues. [‡] Identical across all native genes $i \in \{a, r\}$.

Supplementary Table S2: **Functions and notations appearing in the host cell model ODEs.**

Func./Not.	Description	Formula	Units*
B	Number of translating ribosomes	$R \cdot \frac{D-1}{D}$	nM
T	Proxy for the concentration of the growth-regulating molecule ppGpp	t^c/t^u	None
F_r	Transcription regulation for ribosomal genes (via ppGpp)	$\frac{T}{T+\tau}$	None
	**Ribosome affinities**		
k_i	mRNA-ribosome dissociation constant for gene $i^{\ddagger}$	$\frac{k_i^- + \epsilon/n_i}{k_i^+}$	nM
	**Reaction rates**		
ϵ	Translation elongation rate	$\epsilon_{max} \cdot \frac{t^c}{t^c + K_\epsilon}$	$aa^{\#}/h$
λ	Growth/dilution rate	$\frac{\epsilon B}{M}$	h^{-1}
ψ	tRNA synthesis rate	$\psi_{max} \cdot \frac{T}{T+\tau}$	nM/h
ν	tRNA charging rate	$\nu_{max} \cdot \sigma \cdot \frac{t^u}{t^u + K_\nu}$	h^{-1}

*$E. coli$ volume is $\approx 10^{-18}$ m^3, so 1 nM is roughly equivalent to 1 molecule/cell.

‡ Formula identical across all native **and** synthetic genes $i \in \{a, r\} \cup X$.

#Amino acid residues.

Supplementary Table S3: **Parameters for simulating the expression of the antithetic integral feedback controller circuit.**

Parameter	Description	Value	Units
c_i	Gene DNA concentration*	100	nM
α_{sens}	Sensor gene promoter strength	50	$None$
α_{anti}	Annihilator gene promoter strength	800	$None$
α_{act}	Actuator gene promoter strength	400	$None$
α_{amp}	Amplifier gene promoter strength	$4,000$	$None$
α_{dist}	Disturbing gene promoter strength	500	None
θ	RNA mutual annihilation rate	300	$\frac{1}{nM \cdot h}$
K_{sens}	Half-saturation constant for p_{sens}-annihilator promoter DNA binding	$7,000$	nM
K_{amp}	Half-saturation constant for p_{act}-amplifier promoter DNA binding	700	nM
β_i	mRNA degradation rate*	6	h^{-1}
k_i^+	mRNA-ribosome binding rate‡	60	$\frac{1}{nM \cdot h}$
k_{anti}^+	(Non-translated) annihilator RNA-ribosome binding rate	0	$\frac{1}{nM \cdot h}$
k_i^-	mRNA-ribosome dissociation rate*	60	h^{-1}
n_i	Number of amino acids in protein p_iℵ	300	aa/nM

*Same generic value for all synthetic genes. ‡Same generic value for all synthetic genes except for the annihilator gene *anti*, as it is not translated.
ℵSame generic value for all synthetic genes. For the annihilator gene *anti*, which is not translated, defined as the number of base triplets in the transcript.

References

(1) Hiscock, T. W. Adapting machine-learning algorithms to design gene circuits. *BMC Bioinformatics* **2019**, *20*, 214.

(2) Ma, W.; Trusina, A.; El-Samad, H.; Lim, W. A.; Tang, C. Defining Network Topologies that Can Achieve Biochemical Adaptation. *Cell* **2009**, *138*, 760–773, Publisher: Elsevier.

(3) Sechkar, K.; Steel, H.; Perrino, G.; Stan, G.-B. A coarse-grained bacterial cell model for resource-aware analysis and design of synthetic gene circuits. *Nature Communications* **2024**, *15*, 1981, Publisher: Nature Publishing Group.

(4) Liao, C.; Blanchard, A. E.; Lu, T. An integrative circuit–host modelling framework for predicting synthetic gene network behaviours. *Nature Microbiology* **2017**, *2*, 1658–1666, Publisher: Nature Publishing Group.
